# Online Gambling's Associations With Gambling Disorder and Related Problems in a Representative Sample of Young Swiss Men

**DOI:** 10.3389/fpsyt.2021.703118

**Published:** 2021-07-21

**Authors:** Simon Marmet, Joseph Studer, Matthias Wicki, Yasser Khazaal, Gerhard Gmel

**Affiliations:** ^1^Addiction Medicine, Lausanne University Hospital and University of Lausanne, Lausanne, Switzerland; ^2^Research Centre, University Institute of Mental Health, Montréal, QC, Canada; ^3^Addiction Switzerland, Lausanne, Switzerland; ^4^Centre for Addiction and Mental Health, Toronto, ON, Canada; ^5^Faculty of Health and Social Sciences, University of the West of England, Bristol, United Kingdom

**Keywords:** online gambling, internet gambling, Switzerland, gambling disorder, gambling

## Abstract

**Background and Aims:** Internet gambling has recently grown in popularity, but relatively little is known about how online and the combination of online and offline (mixed) gambling are associated with gambling disorder (GD) and related problems. The present research examined in a cohort study sample of young Swiss men how their gambling activities and gambling-related problems differed across the spectrum from offline to online gambling.

**Sample:** A general-population based sample from the Cohort Study on Substance Use Risk Factors (C-SURF), consisting of 5,352 young Swiss men (mean age 28.26 years old).

**Measures:** The spectrum from exclusively offline to almost exclusively online (>90% of gambling money spent online) gambling was measured using one question about the proportion of gambling money spent online. Total money gambled and time spent on gambling were also assessed. GD severity (range 0–9) was measured using items reflecting the nine DSM-5 GD criteria. The number of gambling-related problems (e.g., financial difficulties, range 0–10), other addictive disorders and mental health problems were also inquired about.

**Methods:** We estimated a generalised linear model using a count model (negative binomial link function) for GD severity and gambling-related problems associated with the amounts and proportions of money gambled online and offline.

**Results:** The number of GD criteria were associated with money gambled online (IRR [95%CI] = 2.81 [2.43, 3.24]) and offline (IRR = 2.68 [2.40, 3.00]). This was also found for the number of gambling-related problems (IRR = 2.43 [2.13, 2.79] and IRR = 2.89 [2.59, 3.23]). Compared with exclusively-offline gamblers, mixed gamblers (26–90% of money gambled online) showed the highest levels of GD symptoms and gambling-related problems, followed by the almost-exclusively-online gamblers (≥91% money gambled online) and, overall, these associations were still significant after adjustment for overall involvement in gambling (time spent and money gambled). Levels of other addictive disorders and mental health problems were higher among mixed gamblers than among offline-only gamblers, but levels among almost-exclusively-online gamblers were not.

**Conclusions:** Symptoms of gambling disorder and gambling related problems are highest among gamblers engaging in both offline and online gambling. Prevention efforts need to target the combination of offline and online gambling.

## Introduction

Gambling is a common leisure activity in Switzerland, with 69.0% of the general adult population being lifetime gamblers and 55.0% having gambled in the last 12 months (1). Classic gambling activities, like lotteries, betting, card games, and casino gambling, have recently been complemented by online gambling activities. The present research used a sample from a large cohort study of young Swiss men to investigate whether the proportion of online gambling activities was associated with symptoms of gambling disorder (GD), gambling-related problems, other addictive disorders and indicators of mental health. The legal gambling situation in Switzerland has evolved in recent decades. Casino gambling (except in games with very small stakes: a maximum of CHF 5) was forbidden from 1877 to 2000, which led to a long tradition of gambling in nearby casinos abroad. Furthermore, Swiss casino operators were not allowed to offer online gambling services until 2019, and these could only be offered by foreign gambling service providers (2, 3). After a decade-long legislative process, the tables have turned: access to online gambling services based outside Switzerland was outlawed in 2019 and, instead, domestic casinos were given licences to offer online gambling services (2). The first domestic online gambling services were launched half a year after the law was implemented (4).

Although most people do not develop any problems due to their gambling activities, some develop symptoms of GD (5). According to the DSM-5 (5), GD is characterised by repeated problematic gambling behaviour resulting in significant problems or distress. It defines nine criteria for the assessment of GD, e.g., the need to gamble with increasing amounts, chasing losses and unsuccessful efforts to control gambling (5). Indeed, GD is the only *behavioural addiction* currently fully recognised by the DSM-5 (5). The ICD-11 also includes a diagnosis for gambling, with specifiers for predominantly online or predominantly offline gambling disorder (6).

Internet gambling has received increasing interest recently. It allows easy access to many different betting options, instant feedback and continuous gambling with large amounts of money. Therefore, concerns are growing that it may pose a particularly high risk for GD and gambling-related problems (7–9). A review by Gainsbury (7) found that numerous studies had reported associations between internet gambling and gambling disorder. However, these associations were often no longer significant once other variables had been controlled for, notably, overall involvement in gambling (time spent or money gambled) and offline (land-based) gambling (7, 10). According to Gainsbury (7), people who engaged in both online and offline gambling appear to have greater risks of experiencing harm, and the relationship between online gambling and gambling problems may be confounded by land-based gambling. Thus, offline gambling activities and overall involvement in gambling are important factors to consider when assessing the risks related to online gambling. Gainsbury (7) concluded that “Internet gambling does not cause gambling problems in, and of, itself,” but internet gambling is more common among highly involved gamblers and may contribute significantly to gambling problems for some of them. A recent study involving more than 9,000 adolescents (10) also concluded that when it came to problem gambling, overall involvement in gambling (time spent and diversity of gambling formats) should be considered rather than internet gambling *per se*. This viewpoint was supported by a study reviewing the gambling policies in 30 European countries. It found that there were no associations between online gambling regulations, gambling licencing systems and legal gambling opportunities, and the prevalence of GD (11). However, a recent study using propensity score matching for offline, online and mixed gamblers found that online gambling alone or in combination with offline gambling posed greater risks to gamblers than offline gambling alone (12).

Gambling is also known to be associated with substance use disorders and behavioural addictions, as well as mental health comorbidities such as major depression or anxiety (13, 14). Regarding the associations between online gambling and mental health comorbidities, findings are heterogeneous across studies (7). A number of studies found higher rates of mental health comorbidities among online gamblers than offline gamblers, whereas others found no such associations. Thus, associations between internet gambling and mental health issues remain unclear (7).

### Aims

Internet gambling is a growing concern, but evidence about its association with GD is somewhat inconclusive. Furthermore, potential associations may actually be evolving quickly in conjunction with the development of new policies and online gambling opportunities and supply. Most of the studies that have assessed associations between online gambling and problem gambling only reported on gamblers categorised as offline, online and mixed gamblers, with no consideration of the proportion of their involvement in online gambling. The present study aimed to address this limitation by asking about the actual proportion of money gambled online. It investigated online vs. offline gambling from two complementary perspectives: one using the amount of money gambled online as a continuous predictor of GD and its related problems, and the other using a categorical approach involving the proportion of total gambling money gambled online.

Specifically, the study's first aim was to test associations between gamblers' involvement in online gambling (using amounts of money gambled online and offline as proxies) and GD symptoms and gambling-related consequences.

The second aim was to investigate online vs. offline gambling from a different approach, testing how GD symptoms and gambling-related consequences differed across the spectrum from offline to almost-exclusively-online gamblers. It would also look at the degree to which these associations were due to greater involvement in mixed and almost exclusively online gambling.

Finally, because findings regarding the associations between online gambling and mental health are relatively few and heterogeneous across studies (7), the study's third aim was to investigate whether other addictive behaviours and indicators of mental health were associated with offline and online gambling in our sample.

## Method

### Sample

Our sample came from the Cohort Study on Substance Use Risk Factors (C-SURF), designed to examine patterns of addictive behaviours and related factors among young Swiss men (15, 16). Enrolment for the baseline assessment in 2010 took place during the recruitment procedures testing fitness for military service, which are compulsory (17) for all young Swiss men, with rare exceptions for those with severe disability, for example. Thus, the sample can be considered to be representative of its source population. Young men were enrolled at three of the six national military recruitment centres (in Lausanne, Windisch and Mels), which cover 21 of Switzerland's 26 cantons. The Human Research Ethics Committee of the Canton of Vaud approved the research protocol for the C-SURF study (protocol 15/07). Overall, 7,556 participants gave their written informant consent to participate at the study after the enrolment procedure, and 5,854 participants were asked to fill out the fourth wave questionnaire on paper or online. A total of 5,368 participants replied to it between April 2019 and November 2020. A sampling flow chart with more details about the study design can be found at https://www.c-surf.ch/en/1.html. Sixteen were excluded because of missing values on main variables, resulting in a sample size of 5,352. Furthermore, 300 participants replied after 14 February 2020. Although their responses may have been affected by the COVID-19 crisis, after careful evaluation, we concluded that the non-response bias introduced by excluding these late-responders [who may differ from early responders; (16)] would have been at least equal to the bias introduced by the COVID-19 crisis and we decided to retain these participants in the sample. As a sensitivity analysis, we provide the main results (Tables 1, 2) without the 300 participants that replied after 14 February 2020 in Supplementary Tables 1, 2.

**Table 1:** Descriptive statistics for the whole sample.

	**Total sample**		**Gamblers only**^**a**^	
	***N***	***%/*mean (SD)**	***N***	**%/mean (SD)**
*N* Total	5,352		1,526	
Age (years)		28.26 (1.27)		28.31 (1.26)
**Linguistic region**
French-speaking	3,111	58.1%	940	61.6%
German-speaking	2,241	41.9%	586	38.4%
**Gambling disorder in the past 12 months**
Gambling disorder score		0.09 (0.58)	1,526	0.32 (1.04)
Gambling disorder prevalence				
No (3 criteria or less)	5,311	99.2%	1,485	97.3%
Yes (4 criteria or more)	41	0.8%	41	2.7%
**Money gambled per month (CHF; past 12 months)**
No gambling	3,826	71.5%		
CHF 1–50	1,134	21.2%	1,134	74.0%
CHF 51–100	196	3.7%	196	12.8%
CHF 100–200	106	2.0%	106	6.9%
CHF 201–500	59	1.1%	59	3.9%
CHF 501–1,000	20	0.4%	20	1.3%
More than CHF 1,000	11	0.2%	11	0.7%
**Mean amount gambled per month (CHF; past 12 months)**
Total		20.74 (91.83)		72.74 (160.63)
Money gambled offline		15.58 (63.96)		54.64 (110.55)
Money gambled online		5.16 (51.79)		18.10 (95.81)
**Proportion of money gambled online (past 12 months)**
No gambling	3,826	71.5%		
Offline gambling only	1,066	19.9%	1,066	69.9%
1–25% online	244	4.6%	244	16.0%
26–50% online	55	1.0%	55	3.6%
51–75% online	45	0.8%	45	2.9%
76–90% online	33	0.6%	33	2.2%
≥91% online	83	1.6%	83	5.4%
**Gambling activities (days per year) in the past 12 months**
Lotteries		3.97 (16.92)		13.91 (29.43)
Electronic lotteries (tactilo)		0.61 (8.41)		2.14 (15.66)
Machines		0.57 (6.24)		1.99 (11.57)
Tables at a casino		1.05 (7.68)		3.69 (14.04)
Internet		1.69 (15.29)		5.95 (28.2)
Private		0.71 (6.17)		2.51 (11.36)
Other		0.46 (7.79)		1.61 (14.53)

a *Gamblers were defined as participants that reported any gambling the past 12 months*.

**Table 2:** Gambling disorder criteria, gambling problems (financial problems, mental stress, problems at work, etc), gambling frequency and money used by proportion of money gambled online (among gamblers, *N* = 1,526).

**Proportion of online gambling**	**Offline gambling only**	**1–25% online**	**26–50% online**	**51–75% online**	**76–90% online**	**≥91% online**
*n*	1,066	244	55	45	33	83
Gambling disorder criteria	0.12	0.56	1.29	1.11	1.52	0.69
Number of gambling problems	0.28	1.11	2.24	1.18	1.33	0.45
Hours of gambling per week	0.26	1.25	1.33	2.18	1.58	1.15
Total money (CHF) gambled per month	47.56	103.28	191.82	150.56	220.45	126.51
Money offline	47.56	89.85	118.93	55.71	37.48	5.69
Money online	0.00	13.43	72.89	94.85	182.98	120.81

### Measures

#### Gambling-Related Measures

Participants that reported any gambling in the last 12 months were considered as gamblers. The frequencies of seven different gambling activities (internet, lotteries, electronic lottery, machines, tables at a casino, private, other) were measured using a tabular question format, with which participants could indicate how often they did these different activities. Response options were “never,” “a few times per year,” “multiple times per month,” “multiple times per week,” and “every day or almost every day.” These answers were recoded into days per year.

Time spent gambling was measured using two questions asking subjects how often (recoded to days per week) they gambled in the last 12 months and how many hours they spent gambling on those days. The product of the answers to these two questions was calculated to estimate the hours spent gambling per week.

Just one question was used to ask the proportion of total money gambled online. Response options were “no money gambled online, only offline,” “1–25% online,” “26–50% online,” “51–75% online,” “76–90% online,” “≥91% online.” The proportion of money gambled was chosen as a proxy for the importance of gambling activities. This agreed with a study showing that online gamblers considered money limits to be one of their most important harm reduction strategies (above time limits) (18). Furthermore, numerous studies have reported on problems related to money and indebtedness among online gamblers (19–21). The proportion of total gambling money gambled online could thus be considered as a relevant proxy for assessing involvement in online gambling.

Money gambled was measured using one question asking subjects how much money they had spent monthly on average on gambling over the last 12 months. Response options were from CHF 1–50 to more than CHF 1,000, and these were recoded to CHF (about EUR 0.9 or USD 1.1) gambled per month.

The approximate amounts of money gambled online and offline were calculated in CHF by multiplying the total amount of money gambled by the proportion of money gambled online, using the following weightings: “only offline” (0% online; 100% offline), “1–25% online” (13% online; 87% offline), “26–50% online” (38% online; 62% offline), “51–75% online” (63% online; 37% offline), “76–90%” online (83% online; 17% offline) and “≥91% online” (95.5% online; 4.5% offline).

The primary outcome was GD severity, which was measured using the nine DSM-5 criteria (5) adapted from (22) in a *yes or no* format and with a total score ranging from 0 to 9.

Gambling-related consequences were measured using 10 questions asking how often subjects had experienced those criteria in the last 12 months (e.g., serious financial consequences for oneself or someone close due to gambling). The four response options were “never,” “rarely,” “sometimes,” or “often,” and these were recoded to *yes or no* for reasons of parsimony after verification that results were similar to if they had been used as continuous scores. These questions were adapted from the Finnish Gambling Harm Survey 2016 (23).

#### Other Addictive Behaviours

Alcohol use disorder over the last 12 months was assessed using 12 *yes or no* (scored 1 and 0, respectively) items representing the 11 DSM-5 alcohol use disorder criteria (5, 24, 25). The sum of the item scores, used for the analysis, ranged from 0 to 11.

Cannabis use disorder was measured using the ten-item Cannabis Use Disorders Identification Test [CUDIT-R (26); revised version of (27)], building a score ranging from 0 to 40.

Tobacco use disorder over the last 12 months was measured using the six-item Fagerström test for nicotine dependence (28, 29), forming a score ranging from 0 to 10.

Internet addiction was measured using the Compulsive Internet Use Scale (CIUS), consisting of 14 five-point Likert scale items (30–33). Summing their results built a score ranging from 0 to 56.

Gaming addiction over the last 6 months was measured using the seven-item (five-point Likert scale) Game Addiction Scale (34, 35), resulting in a score ranging from 0 to 28.

Participants were asked how often over the last 12 months they had used illicit substances (or substances not intended for consumption) from a list of 16 substances: ecstasy, cocaine, heroin, methadone, hallucinogens (multiple), khat, poppers, amphetamines, crystal meth, inhalants or solvents, ketamine, GHB, research chemicals, and spice. Response options were “never,” “1–3 times” (recoded as 2), and “≥4 times” (recoded as 4), and their sum was built into an approximate frequency of illicit drug use, with a score capped at 20.

#### Mental Health Indicators

Symptoms of social anxiety disorder (SAD) during the past week were assessed using the Clinically Useful Social Anxiety Disorder Outcome Scale (CUSADOS), measured via 12 five-point Likert scale items, forming a score ranging from 0 to 48.

Life satisfaction was assessed using the Satisfaction with Life Scale (36), consisting of five items with response options from 1 (strongly disagree) to 7 (strongly agree). The sum of the items ranged from 5 to 35.

The severity of major depression over the last 2 weeks was assessed using the Major Depression Inventory [WHO–MDI; (37, 38)], consisting of 12 items on a six-point Likert scale and used to form 10 criteria and a score ranging from 0 to 50.

### Statistical Analysis

Descriptive statistics were calculated for the overall sample and by category of the proportion of money gambled online. For Aim 1, negative binomial regressions were used to test associations between the outcomes' GD criteria and gambling-related problems. In a first step, this was done bivariately, and in a second step, amounts of both online and offline money were entered into the regression model together. The resulting coefficients were multiplied by 100 and then log transformed to get an incidence rate ratio (IRR) per CHF 100 gambled (for better readability) and a *beta* per CHF 100 gambled for the linear regression models for other addictive disorders and indicators for mental health. For Aim 2, differences in GD symptoms and gambling-related problems across the spectrum from offline to online gambling were tested using negative binomial regressions, with offline-only gamblers being the reference group. IRRs are reported for negative binomial regressions. In a second step, these analyses were adjusted for the time spent and money gambled to account for differences in involvement in gambling. The prevalence for each of the 10 gambling related problems and of reporting any of the 10 problems was calculated separately for each category of the proportion of money spent online. Chi-square tests were performed to test whether these individual problems differed significantly across the spectrum from offline to online gambling. For Aim 3, the analyses made for amounts of money gambled online and offline (as in Aim 1) and for the spectrum from offline to online gambling (as in Aim 2) were repeated for addictive disorders (using negative binomial regressions) and mental health indicators (linear regressions). All analyses were adjusted for age and linguistic region (French vs. German) and carried out using SPSS 25 software.

## Results

Descriptive statistics are presented in Table 3. In the past year, 28.5% of the sample had gambled. The most frequent gambling activity was playing lotteries (3.97 days per year on average), followed by internet gambling (1.69 days per year). About 20% of the sample only gambled offline, 4.6% mostly gambled offline (1–25% of total money spent on gambling gambled online), 2.4% were mixed gamblers (25–90% of money gambled online), and 1.6% were almost-exclusively-online gamblers (≥91% of money gambled online). Of the total sample, 0.8% showed 4 or more DSM-5 GD symptoms, corresponding to 2.7% of gamblers.

**Table 3:** Negative binomial regression (IRR [95% CI]) on gambling disorder symptoms, gambling problems, other addictive disorders and mental health variables by money (per CHF 100) gambled online and offline.

	**Bivariate**		**Multivariable**	
	**Online**	**Offline**	**Online**	**Offline**
**Gambling disorder and related problems (negative binomial count regression; IRR [95% CI])**				
Gambling disorder criteria	**2.81 [2.43, 3.24]**	**2.68 [2.40, 3.00]**	**1.87 [1.66, 2.12]**	**2.04 [1.82, 2.29]**
Gambling-related problems	**2.43 [2.13, 2.79]**	**2.89 [2.59, 3.23]**	**1.50 [1.35, 1.67]**	**2.44 [2.18, 2.72]**
**Addictive disorders (negative binomial count regression; IRR [95% CI])**				
Alcohol use disorder	**1.09 [1.02, 1.17]**	**1.11 [1.05, 1.17]**	1.05 [0.98, 1.13]	**1.09 [1.03, 1.16]**
Cannabis use disorder	1.06 [0.98, 1.16]	**1.12 [1.06, 1.18]**	0.97 [0.89, 1.06]	**1.13 [1.06, 1.20]**
Tobacco use disorder	**1.17 [1.10, 1.26]**	**1.21 [1.14, 1.30]**	**1.11 [1.04, 1.19]**	**1.17 [1.10, 1.25]**
Illicit drug use	**1.19 [1.10, 1.30]**	**1.17 [1.11, 1.24]**	**1.10 [1.01, 1.19]**	**1.14 [1.07, 1.21]**
Gaming addiction	**1.17 [1.08, 1.26]**	**1.12 [1.06, 1.17]**	**1.10 [1.02, 1.20]**	**1.08 [1.02, 1.14]**
Internet addiction	**1.12 [1.05, 1.20]**	**1.04 [1.00, 1.09]**	**1.11 [1.04, 1.20]**	1.01 [0.96, 1.06]
**Mental health indicators (linear regression; b [95% CI])**				
Major depression	**0.55 [0.14, 0.95]**	**0.47 [0.13, 0.80]**	**0.43 [0.02, 0.85]**	**0.38 [0.03, 0.72]**
Social anxiety disorder	**0.58 [0.16, 1.00]**	**0.63 [0.28, 0.97]**	0.41 [-0.02, 0.85]	**0.54 [0.19, 0.89]**
Life satisfaction	**-0.49 [-0.82,−0.17]**	**-0.43 [-0.70,−0.17]**	**-0.38 [-0.72,−0.05]**	**-0.36 [-0.63,−0.09]**

*Bold coefficients are significant at p-value < 0.05. Adjusted for age and linguistic region. Bivariate analyses are only adjusted for age and linguistic region, with separate models for money gambled online and offline. In multivariable analyses, money gambled online and offline were entered into the model simultaneously*.

### Amount of Money Gambled Online and Offline

Table 1 shows the results of regression analyses for GD criteria and gambling-related problems as predicted by money gambled online and offline. The number of GD criteria was associated, in a similar magnitude, with money gambled online (IRR [95% CI] = 2.81 [2.43, 3.24]) and offline (IRR = 2.68 [2.40, 3.00]) and with gambling-related problems (IRR = 2.43 [2.13, 2.79] and IRR = 2.89 [2.59, 3.23]). These associations were somewhat attenuated if the amounts of money gambled online and offline were entered into the same model, indicating that they both contributed to some degree to GD and related problems in the same individuals. However, they both contributed significantly in the multivariate models. As regards addictive disorders and mental health indicators (except for cannabis use disorder), amounts of money gambled online and offline were significantly associated with higher levels of addictive disorders and mental health problems, and with lower levels of life satisfaction. As a sensitivity analysis, we provided results for Tables 1, 2 without the 300 participants (of which 92 actually gambled in the last 12 months) that replied after 14 February 2020 in Supplementary Tables 1, 2. Overall, for the outcomes gambling disorder and gambling related problems, coefficients were slightly higher without these 300 participants, while they tended to be slightly lower (in some cases just below significance) for substance use disorders and mental health outcomes.

### Differences Across Groups From Offline to Online Gamblers

**Figure 1:**
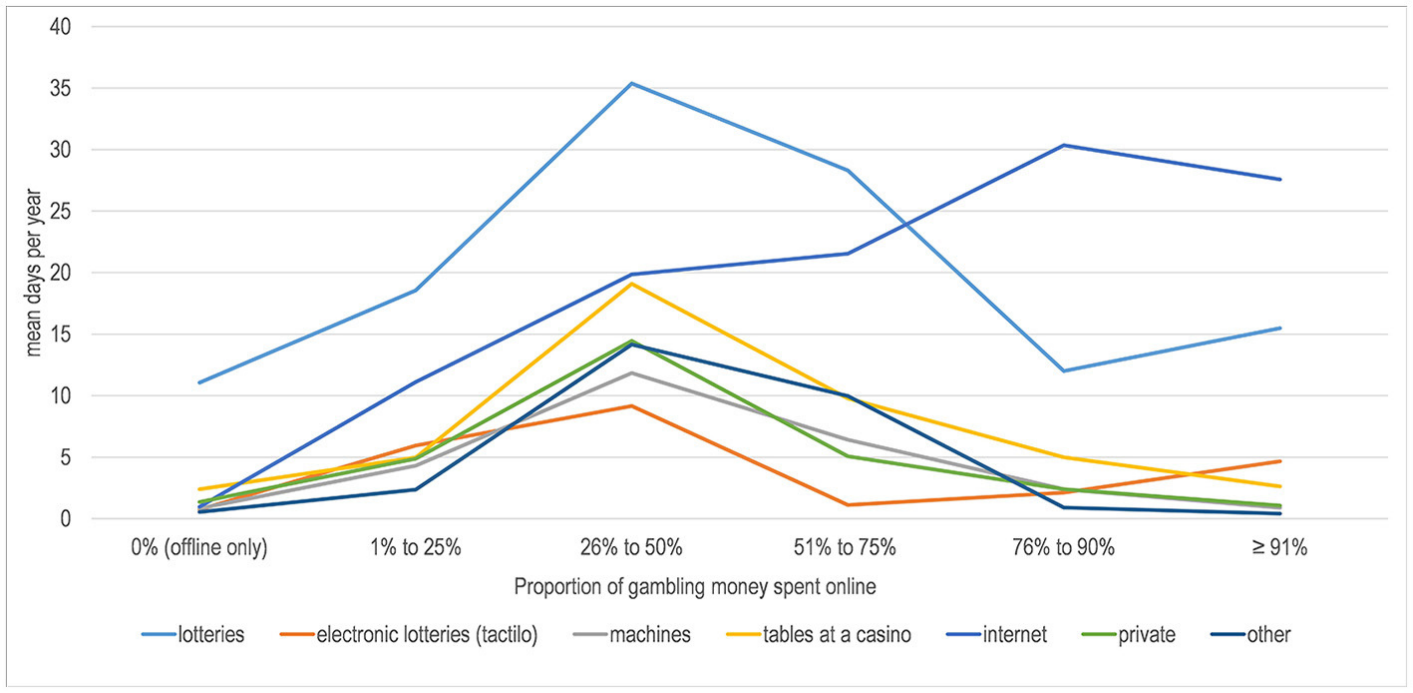
Gambling activities (days per year in the last 12 months) by proportion of money gambled online.

Hours per week spent gambling and money gambled per year were lowest in the offline gambling group, peaked in the mixed group and were again a bit lower in the almost exclusively online gambling group (Table 4). Figure 1 shows seven gambling activities across the spectrum from offline to online gambling. The most frequent gambling activity among offline gamblers was playing lotteries. The most frequent activity among mixed gamblers was also playing lotteries, but other activities such as playing tables at a casino were also more frequent than among offline-only gamblers. Among the almost exclusively online gambling group, playing lotteries was the only other somewhat regular gambling activity, with gambling at a casino or on machines being quite rare in this group.

**Table 4:** Negative binomial regression (IRR [95% CI]) on gambling disorder criteria, gambling problems, other addictive disorders and mental health variables by proportion of money gambled online.

	**Non-gambler**	**Offline only**	**1–25% online**	**26–50% online**	**51–75% online**	**76–90% online**	**≥91% online**
***n***	**3,826**	**1,066**	**244**	**55**	**45**	**33**	**83**
**Gambling disorder criteria (negative binomial count regression)**							
Unadjusted	n.a.	ref.	**4.77 [3.60, 6.32]**	**11.46 [7.67, 17.11]**	**9.47 [6.06, 14.82]**	**13.12 [8.10, 21.24]**	**6.10 [4.14, 9.00]**
Adjusted for time spent and money gambled	n.a.	ref.	**3.83 [2.87, 5.13]**	**6.74 [4.39, 10.35]**	**6.21 [3.87, 9.96]**	**6.12 [3.56, 10.50]**	**3.80 [2.48, 5.81]**
**Gambling-related problems (negative binomial count regression)**							
Unadjusted	n.a.	ref.	**3.95 [3.17, 4.90]**	**8.29 [5.87, 11.70]**	**4.20 [2.76, 6.39]**	**4.89 [3.05, 7.84]**	**1.60 [1.06, 2.42]**
Adjusted for time spent and money gambled	n.a.	ref.	**3.43 [2.75, 4.28]**	**5.17 [3.58, 7.46]**	**2.56 [1.62, 4.06]**	**2.15 [1.25, 3.70]**	0.90 [0.56, 1.45]
**Addictive disorders (negative binomial count regression)**							
Alcohol use disorder	**0.76 [0.69, 0.83]**	ref.	1.07 [0.89, 1.29]	**1.56 [1.12, 2.17]**	1.21 [0.83, 1.78]	1.36 [0.88, 2.10]	0.96 [0.71, 1.30]
Cannabis use disorder	**0.83 [0.76, 0.90]**	ref.	1.10 [0.93, 1.31]	**2.11 [1.55, 2.88]**	0.80 [0.54, 1.19]	1.19 [0.78, 1.81]	1.14 [0.86, 1.50]
Tobacco use disorder	**0.77 [0.70, 0.84]**	ref.	**1.28 [1.07, 1.54]**	**1.61 [1.14, 2.27]**	1.04 [0.69, 1.57]	1.23 [0.77, 1.95]	0.91 [0.66, 1.25]
Illicit drug use	0.93 [0.84, 1.02]	ref.	**1.84 [1.54, 2.19]**	**2.96 [2.16, 4.05]**	**1.57 [1.07, 2.28]**	0.51 [0.28, 0.90]	1.17 [0.87, 1.59]
Gaming addiction	1.02 [0.94, 1.10]	ref.	**1.37 [1.16, 1.61]**	**2.12 [1.57, 2.87]**	**1.75 [1.25, 2.44]**	**1.60 [1.08, 2.37]**	1.21 [0.94, 1.58]
I nternet addiction	0.98 [0.91, 1.06]	ref.	1.11 [0.96, 1.28]	**1.51 [1.14, 2.01]**	**1.39 [1.02, 1.89]**	**1.66 [1.16, 2.38]**	**1.28 [1.02, 1.62]**
**Mental health indicators (linear regression)**							
Major depression	−0.15 [−0.69, 0.39]	ref.	**1.11 [0.01, 2.20]**	**4.09 [1.98, 6.21]**	1.21 [−1.14, 3.56]	2.18 [−0.52, 4.88]	1.09 [−0.65, 2.83]
Social anxiety disorder	−0.07 [−0.62, 0.48]	ref.	**2.10 [0.98, 3.23]**	**5.16 [2.99, 7.33]**	**3.40 [0.99, 5.82]**	**4.18 [1.41, 6.96]**	0.18 [−1.61, 1.97]
Life satisfaction	0.28 [−0.15, 0.71]	ref.	**−1.33 [−2.20,−0.46]**	**−2.48 [−4.18**, **−0.79]**	−1.39 [−3.26, 0.48]	−1.85 [−4.02, 0.32]	−0.70 [−2.10, 0.69]

*Bold coefficients are significant at p-value < 0.05. Adjusted for age and linguistic region. N.a., not applicable because not assessed in non-gamblers*.

Compared to exclusively-offline gamblers, numbers of GD symptoms were significantly higher among mostly-offline gamblers (1–25% gambling money spent online; IRR = 4.77 [3.60, 6.32]), mixed gamblers (26–50%: IRR = 11.46 [7.67, 17.11]; 51–75%: IRR = 9.47 [6.06, 14.82]; 76–90%: IRR = 13.12 [8.10, 21.24]), and almost-exclusively-online gamblers (IRR = 6.10 [4.14, 9.00]), with the peak being among mixed gamblers (see Table 4 for means and Table 2 for regression results). These coefficients were attenuated after adjustment for involvement in gambling (time spent and money gambled) but nevertheless remained high and significant. Results for numbers of gambling-related problems were similar, but the coefficient for almost-exclusively-online gamblers was no longer significant after adjustment for the time spent and money gambled. Individuals' gambling-related problems showed a similar pattern overall: they were lowest among offline gamblers, highest among mixed gamblers and in-between among almost-exclusively-online gamblers (≥91% money gambled online) (Table 5). Differences across categories of proportion of money spent online were significant for all 10 individual gambling related problems. The most frequently reported problems among almost-exclusively-online gamblers were reduced performance at school or work (8.4%), sleep problems (7.2%), serious financial problems for oneself (8.4%) and mental stress (7.7%). In contrast, interpersonal problems and serious financial problems for someone else were reported relatively rarely.

**Table 5:** Specific gambling-related problems (proportion at least once in the last 12 months) by proportion of money gambled online, and chi-square tests for overall differences across proportion of money spent online (among gamblers, *n* = 1,526).

	**Offline gambling only**	**1–25% online**	**26–50% online**	**51–75% online**	**76–90% online**	**≥91% online**	**Total all gamblers**	**Chi-square (df = 5)**	***P*-value**
*n*	1,066	244	55	45	33	83	1,526		
Serious financial problems	3.3%	12.9%	21.8%	13.6%	24.2%	8.4%	6.6%	75.74	<0.001
Serious financial problems for someone close	3.4%	10.4%	23.6%	13.6%	12.1%	1.2%	5.6%	64.82	<0.001
Mental stress (depression, anxiety, etc.)	3.1%	13.3%	25.5%	13.6%	15.2%	7.2%	6.4%	80.14	<0.001
Relationship problems (with partner, family)	2.9%	12.1%	18.2%	13.6%	18.2%	1.2%	5.5%	69.40	<0.001
Serious health problems or injury	2.3%	9.6%	18.2%	9.1%	3.0%	1.2%	4.2%	57.46	<0.001
Serious problems at work or school	1.9%	8.3%	18.2%	11.4%	9.1%	2.4%	4.0%	60.80	<0.001
Reduced performance at work or school	2.3%	10.8%	20.0%	15.9%	12.1%	8.4%	5.3%	71.35	<0.001
Sleep problems	2.6%	9.6%	23.6%	11.4%	15.2%	7.2%	5.3%	70.92	<0.001
Increased tobacco use	4.2%	14.2%	30.9%	9.1%	15.2%	4.8%	7.2%	80.93	<0.001
Increased alcohol use	2.9%	11.3%	23.6%	9.1%	9.1%	2.4%	5.3%	69.30	<0.001
Any of the above problems	9.8%	28.3%	47.3%	24.4%	30.3%	18.1%	15.4%	108.92	<0.001

*Questions were phrased as “How often did gambling games cause these problems in the last 12 months?”*.

Regarding more distal correlates, offline gamblers showed significantly higher levels of alcohol, cannabis, and tobacco use disorder than non-gamblers, but not for illicit drug use, gaming and internet addiction, nor for indicators of mental health (Table 5). Compared to offline gamblers, there was a general tendency for mixed gamblers to show higher levels of addictive disorders, depression and social anxiety disorder; they also showed lower life satisfaction, but this did not reach significance in all categories of mixed gamblers. Almost-exclusively-online gamblers (≥91% of money gambled online) generally showed few differences from offline-only gamblers, and this only reached significance for internet addiction.

## Discussion

The present study had three aims. First, to analyse the association between online and offline gambling involvement and GD symptoms and gambling-related problems. Second, to look at groups of gamblers according to their proportion of online and offline gambling on GD and problems, and third, to look at the associations between on- and offline gambling with other addictive behaviours and mental health.

Overall, our participants spent about three times as much gambling money offline than online. Two thirds of gamblers (69.9%; 19.9% of the total sample) gambled exclusively offline, and this group spent considerably less time gambling and gambled less money than those who also played online. Mixed gamblers (1–90% of gambling money spent online) represented 7.0% of the total sample (30.1% of gamblers), whereas almost-exclusively-online gamblers (≥91% of money gambled online) represented about 1.6% (5.4% of gamblers).

As regards our first aim, we found that both the amount of money spent on- and offline were associated with GD and gambling-related problems. These findings were consistent with a recent meta-analysis that found that both internet gambling and offline gambling activities were strong risk factors for problem gambling (39).

As regards our second aim we categorised participants into groups according to the proportion of money they gambled online and compared them (offline, mixed and almost-exclusively-online gamblers) with respect to gambling-related problems. Mixed gamblers spent a lot more time and more money on gambling than exclusively offline gamblers, and they showed higher levels of GD criteria and gambling-related problems. However, almost-exclusively-online gamblers (≥91% of money gambled online) fell in-between offline and mixed gamblers as regards time spent, money gambled, GD criteria and gambling-related problems. Thus, there appeared to be an inverse-U shaped association across the spectrum from offline to online gambling and gambling-related problems, with problems peaking among mixed gamblers. These findings were in line with the review by Gainsbury (7) and some newer studies (12, 40) reporting that gambling-related problems were highest in mixed gamblers. However, Gainsbury (7) conclusion that online gambling may be mainly related to GD through the greater involvement in gambling seen among online gamblers was only partly consistent with our results. In our first approach—the multivariate analysis of the amount of money gambled online and offline—online gambling remained a significant predictor of GD and gambling-related problems, even after adjustment for offline gambling. In our second approach, the differences between groups of gamblers, ranging from offline to online gamblers, were still significant after adjustment for involvement in gambling (money gambled and time spent), except for the almost-exclusively-online gamblers with respect to gambling-related problems. Thus, our results were partially consistent with earlier findings (7, 10) in that the association of internet gambling and GD is in part due to overall involvement in gambling. However, our study revealed that involvement in online gambling remained an important factor even after adjustments for money gambled offline and overall involvement in gambling. Thus, online gambling is a risk factor for GD, especially when combined with offline gambling.

Regarding specific gambling-related problems, it is noteworthy that almost-exclusively-online gamblers reported interpersonal problems and financial problems for someone close rarely especially compared to mixed gamblers. A possible explanation for this is that online gambling can more easily be kept secret from one's entourage and may be less noticeable; it may therefore create fewer interpersonal conflicts, especially for young men who have fewer social roles and responsibilities than older adults. In line with these findings about interpersonal conflicts, almost-exclusively-online gamblers did not have higher levels of social anxiety disorder than offline gamblers, whereas mixed gamblers did. This is particularly remarkable because one might expect individuals with higher levels of social anxiety disorder to tend to engage in solitary gambling activities online (41). However, based on our data, it could be hypothesised that mixed gamblers more often encounter interpersonal conflict, leading to greater feelings of shame in social interactions and thus symptoms of social anxiety disorder, whereas almost-exclusively-online gamblers report less interpersonal conflict and fewer symptoms of social anxiety disorder.

As regards our third aim, compared to offline gamblers, our sample's mixed gamblers (especially those gambling 26–50% of their money online) reported higher levels of other addictive disorders (alcohol, cannabis, tobacco, illicit drug use, gaming, and internet), major depression and social anxiety disorder, and they also showed lower life satisfaction. Almost-exclusively-online gamblers, however, only showed a significantly higher level of internet addiction, which is unsurprising given that online gamblers probably spend more time on the internet than offline gamblers.

Overall, both online and offline gambling are associated with gambling disorder, gambling-related problems, other addictive disorders and mental health problems. Compared to offline-only gamblers, gamblers engaging in both offline and online gambling appeared to be at a higher risk not only of GD and gambling-related problems but also of other addictive disorders and mental health problems. To date, findings in the literature about associations between gambling and mental health comorbidities have been heterogeneous, with some studies finding an increased risk for mental health comorbidities in online gamblers, while others did not (7). Thus, our findings add one more piece of evidence to the existing literature and point to the importance of considering subjects' degree of involvement in online and offline gambling when investigating associations between online gambling and mental health.

### Limitations

Although our sample only included young Swiss men, young men are a group with a high risk of gambling-related problems. Our general population-based sample provided a different perspective from surveys among gamblers only. The case numbers of individuals with serious gambling problems were small, therefore any conclusions applicable to clinical practise should be done so with great care. Our study did not include detailed measurements of the precise gambling activities engaged in online and offline or the amounts of money gambled and time spent on those individual activities. Such information would be valuable to gain a better understanding of which specific gambling activities were most associated with gambling-related problems. Furthermore, the cross-sectional nature of our results precludes any inference as to the direction of causality, i.e., whether online gambling causes problem gambling or whether gamblers with existing problems tend to use online gambling more often as it is readily available. Finally, about 5% of our sample were late responders, replying to our survey after the onset of the COVID-19 crisis in Switzerland in February 2020, which may have affected their gambling behaviour. However, the time frame for the questions asked was “in the last 12 months,” and we provided a sensitivity analysis without these 300 participants and the results were overall similar, however, in some cases, coefficients were no longer significant without these 300 participants. Overall, the inclusion of these late responders did not alter the conclusions drawn from our study. We decided to use the full sample because excluding late responders (who may differ from early responders) (16) may introduce another type of bias.

### Conclusion

We used two complementary analytical approaches to investigate the associations between involvement in online gambling and gambling-related problems in a large general-population sample of young Swiss men. In our first approach (Aim 1), online gambling and offline gambling both contributed to gambling disorder symptoms and gambling-related problems, and both were associated with other addictive disorders and mental health problems. Our second approach (Aim 2) showed that the peak involvement in gambling, gambling-related problems and mental health comorbidities (Aim 3) was among mixed gamblers. Thus, it appears that the combination of offline and online gambling is associated with most gambling-related problems. Prevention efforts should address both online and offline gambling, but they should also consider interactions between these two domains of gambling. Apart from their risks, online gambling environments may also provide good opportunities to promote responsible gambling using tools that can be personalised to the individual gambler (7). It could also be an environment in which to develop and offer a wide range of gambling-related harm-reduction strategies (18). From a public health perspective, it will be important to monitor further developments in online and offline gambling and to adapt future policies to reduce the impact of online and offline gambling on public mental health.

## Data Availability Statement

The raw data supporting the conclusions of this article will be made available by the authors, without undue reservation.

## Ethics Statement

The studies involving human participants were reviewed and approved by the Human Research Ethics Committee of the Canton of Vaud. The participants provided their written informed consent to participate in this study.

## Author Contributions

SM contributed to the questionnaire design, conducted the data analysis, and wrote the initial draft of the manuscript. MW, GG, JS, and YK contributed to the questionnaire design, data analysis plan, interpretation of the results, and the writing of the manuscript. GG was responsible for the development of the questionnaire and supervised the data collection, and the writing of the manuscript. All authors approved the final version of the manuscript.

## Conflict of Interest

The authors declare that the research was conducted in the absence of any commercial or financial relationships that could be construed as a potential conflict of interest.
