# Supplementary material for: Online Gambling's Associations With Gambling Disorder and Related Problems in a Representative Sample of Young Swiss Men
Source: Front Psychiatry. 2021 Jul 21;12:703118. doi: 10.3389/fpsyt.2021.703118 (PMC8335561; doi:10.3389/fpsyt.2021.703118)
Supplement: Supplementary file 1 [file Data_Sheet_1.pdf]

Online gambling's associations with gambling disorder and related problems in a representative sample of young Swiss men

Simon Marmet[1], Joseph Studer[1], Matthias Wicki[1], Yasser Khazaal[1,2] and Gerhard Gmel[1, 3, 4, 5]

1 Addiction Medicine, Lausanne University Hospital and University of Lausanne, Rue du Bugnon 23, CH-1011 Lausanne, Switzerland
2 Research Centre, University Institute of Mental Health at Montréal, Québec, Canada
3 Addiction Switzerland, Avenue Louis-Ruchonnet 14, CH-1001 Lausanne, Switzerland
4 Centre for Addiction and Mental Health, 1001 Queen Street West, Toronto ON - M6J 1H4, Canada
5 University of the West of England, Frenchay Campus, Coldharbour Lane, Bristol BS16 1QY, United Kingdom

Corresponding author's email: simon.marmet@chuv.ch

Table S1. Negative binomial regression (IRR [95% CI]) on gambling disorder symptoms, gambling problems, other addictive disorders and mental health variables by money (per CHF 100) gambled online and offline. **Only participants (n=5055) that replied before 14 February 2020 (pre-COVID).**

	**bivariate**		**multivariable**	
	online	*offline*	*online*	*offline*
**Gambling disorder and related problems (negative binomial count regression; IRR [95% CI])**				
Gambling disorder criteria	**2.93 [2.50, 3.42]**	**2.74 [2.43, 3.08]**	**1.92 [1.68, 2.19]**	**2.07 [1.83, 2.34]**
Gambling-related problems	**2.46 [2.12, 2.85]**	**2.99 [2.65, 3.36]**	**1.51 [1.36, 1.69]**	**2.52 [2.24, 2.84]**
**Addictive disorders (negative binomial count regression; IRR [95% CI]))**				
Alcohol use disorder	**1.08 [1.01, 1.16]**	**1.09 [1.03, 1.16]**	1.05 [0.98, 1.13]	**1.08 [1.02, 1.15]**
Cannabis use disorder	0.95 [0.87, 1.04]	1.04 [0.98, 1.11]	0.93 [0.84, 1.03]	1.06 [0.99, 1.13]
Tobacco use disorder	**1.16 [1.08, 1.25]**	**1.22 [1.13, 1.31]**	**1.12 [1.04, 1.19]**	**1.18 [1.10, 1.27]**
Illicit drug use	**1.13 [1.04, 1.23]**	**1.14 [1.07, 1.22]**	1.08 [1.00, 1.17]	**1.12 [1.05, 1.20]**
Gaming addiction	**1.13 [1.04, 1.22]**	**1.09 [1.03, 1.15]**	**1.09 [1.01, 1.18]**	**1.07 [1.01, 1.13]**
Internet addiction	**1.10 [1.03, 1.18]**	1.03 [0.98, 1.08]	**1.10 [1.02, 1.17]**	1.01 [0.96, 1.06]
**Mental health indicators (linear regression; b [95% CI])**				
Major depression	0.36 [-0.05, 0.78]	0.34 [-0.01, 0.69]	0.29 [-0.13, 0.71]	0.28 [-0.07, 0.64]
Social anxiety disorder	0.34 [-0.09, 0.76]	**0.41 [0.05, 0.76]**	0.24 [-0.19, 0.68]	0.36 [-0.01, 0.73]
Life satisfaction	**-0.48 [-0.81, -0.15]**	**-0.43 [-0.71, -0.16]**	**-0.39 [-0.72, -0.05]**	**-0.36 [-0.64, -0.08]**

Notes: bold coefficients are significant at *p*-value < 0.05. Adjusted for age and linguistic region. Bivariate analyses are only adjusted for age and linguistic region, with separate models for money gambled online and offline. In multivariable analyses, money gambled online and offline were entered into the model simultaneously.

Table S2. Negative binomial regression (IRR [95% CI]) on gambling disorder criteria, gambling problems, other addictive disorders and mental health variables by proportion of money gambled online. **Only participants (n=5055) that replied before 14 February 2020 (pre-COVID).**

	non-gambler	offline only	1% to 25% online	26% to 50% online	51% to 75% online	76% to 90% online	≥ 91% online
n	3826	1066	244	55	45	33	83
**Gambling disorder criteria (negative binomial count regression)**							
Unadjusted	n.a.	ref.	**5.01 [3.73, 6.72]**	**11.60 [7.57, 17.79]**	**9.71 [6.03, 15.63]**	**13.72 [8.44, 22.30]**	**6.54 [4.40, 9.70]**
Adjusted for time spent and money gambled	n.a.	ref.	**3.97 [2.93, 5.39]**	**7.45 [4.75, 11.68]**	**6.52 [3.95, 10.75]**	**6.52 [3.79, 11.23]**	**4.09 [2.66, 6.30]**
**Gambling-related problems (negative binomial count regression)**							
Unadjusted	n.a.	ref.	**4.30 [3.44, 5.39]**	**7.68 [5.30, 11.14]**	**3.75 [2.37, 5.95]**	**5.08 [3.16, 8.16]**	**1.71 [1.13, 2.59]**
Adjusted for time spent and money gambled	n.a.	ref.	**3.71 [2.95, 4.67]**	**5.19 [3.52, 7.67]**	**2.23 [1.34, 3.72]**	**2.26 [1.31, 3.91]**	0.96 [0.60, 1.56]
**Addictive disorders (negative binomial count regression)**							
Alcohol use disorder	**0.76 [0.70, 0.84]**	ref.	1.10 [0.91, 1.33]	**1.51 [1.06, 2.16]**	1.32 [0.89, 1.95]	1.36 [0.88, 2.11]	0.99 [0.73, 1.34]
Cannabis use disorder	**0.84 [0.77, 0.92]**	ref.	1.18 [0.98, 1.41]	**1.57 [1.11, 2.21]**	0.90 [0.60, 1.35]	1.23 [0.80, 1.88]	1.21 [0.91, 1.60]
Tobacco use disorder	**0.77 [0.70, 0.85]**	ref.	**1.27 [1.05, 1.54]**	1.38 [0.95, 2.01]	1.13 [0.74, 1.74]	1.27 [0.80, 2.02]	0.89 [0.64, 1.24]
Illicit drug use	0.99 [0.91, 1.08]	ref.	**1.29 [1.09, 1.53]**	**1.93 [1.40, 2.65]**	**1.82 [1.28, 2.57]**	**1.58 [1.07, 2.34]**	1.18 [0.91, 1.54]
Gaming addiction	0.97 [0.90, 1.05]	ref.	1.10 [0.95, 1.28]	**1.37 [1.02, 1.84]**	1.33 [0.96, 1.85]	**1.66 [1.16, 2.37]**	**1.28 [1.01, 1.62]**
Internet addiction	0.97 [0.88, 1.07]	ref.	**1.97 [1.64, 2.36]**	**2.50 [1.78, 3.52]**	**1.81 [1.22, 2.67]**	0.53 [0.30, 0.95]	1.27 [0.93, 1.71]
**Mental health indicators (linear regression)**							
Major depression	-0.12 [-0.67, 0.42]	ref.	1.06 [-0.06, 2.19]	**3.13 [0.90, 5.35]**	0.44 [-1.99, 2.86]	2.23 [-0.46, 4.92]	1.14 [-0.62, 2.89]
Social anxiety disorder	0.01 [-0.55, 0.57]	ref.	**2.23 [1.07, 3.39]**	**3.81 [1.52, 6.10]**	**2.87 [0.38, 5.37]**	**4.25 [1.48, 7.02]**	0.27 [-1.54, 2.07]
Life satisfaction	0.25 [-0.18, 0.69]	ref.	**-1.34 [-2.23, -0.44]**	**-1.88 [-3.67, -0.10]**	-1.48 [-3.43, 0.46]	-1.90 [-4.06, 0.25]	-0.89 [-2.30, 0.51]

Note: bold coefficients are significant at p-value < 0.05. Adjusted for age and linguistic region. N.a.: not applicable because not assessed in non-gamblers
